# Enhancing Trial Design for Atypical AD

**DOI:** 10.1002/alz70859_098331

**Published:** 2025-12-25

**Authors:** Paul S. Aisen

**Affiliations:** ^1^ Alzheimer's Therapeutic Research Institute, Keck School of Medicine, University of Southern California, San Diego, CA USA

## Abstract

This session will propose key considerations for advancing clinical trials targeting atypical Alzheimer’s disease (AD) variants. AD variants include early‐onset disease as well as atypical clinical presentations such as prominent language or visuospatial dysfunction. For therapeutic trials, variants may be evaluated singly or combined. Essential trial design elements include selection of populations (demographic, clinical, cognitive and biomarker criteria), selection of candidate treatments, inclusion and exclusion criteria, primary and secondary outcomes, biofluid markers and randomization schemes. Inclusion of syndrome‐specific neuroimaging assessments will be considered. Other issues include statistical considerations such as trial length, sample size determination and analysis plans. The advantages and challenges of platform trial designs for AD variants will be discussed. The session will conclude with a strategic overall approach to clinical trials that can establish effective treatments for those affected by atypical AD variants.

